# Supplementary material for: NECAPs are negative regulators of the AP2 clathrin adaptor complex
Source: eLife. 2018 Jan 18;7:e32242. doi: 10.7554/eLife.32242 (PMC5785209; doi:10.7554/eLife.32242)
Supplement: Supplementary file 1. [file elife-32242-supp1.docx]

**Supplementary File 1**

**A. Strains**

Wild type

Bristol N2

*fcho-1* suppressor screen

| GUN109 | *fcho-1(ox477::unc-119(+)) II ncap-1(mew31[splice donor]) II* |
| --- | --- |
| GUN110 | *fcho-1(ox477::unc-119(+)) II ncap-1(mew32[A29D]) II* |
| GUN111 | *fcho-1(ox477::unc-119(+)) II ncap-1(mew33[S84N]) II* |
| GUN112 | *fcho-1(ox477::unc-119(+)) II ncap-1(mew34[splice donor]) II* |
| GUN113 | *fcho-1(ox477::unc-119(+)) II ncap-1(mew35[splice donor]) II* |
| GUN114 | *fcho-1(ox477::unc-119(+)) II ncap-1(mew36[Q107X]) II* |
| GUN115 | *fcho-1(ox477::unc-119(+)) II ncap-1(mew37[splice donor]) II* |
| GUN116 | *fcho-1(ox477::unc-119(+)) II ncap-1(mew38[stop lost]) II* |
| GUN101 | *fcho-1(ox477::unc-119(+)) II ncap-1(mew39[1.4kb deletion]) II* |

Single-copy transgenes (MosSCIs)

RFP-tagged NECAPs

| GUN118 | *mewSi2[Pdpy-30::RFP:NCAP1 unc-119(+)] II; unc-119(ed3) III* |
| --- | --- |
| GUN73 | *mewSi3[Pdpy-30::RFP:NCAP-1 unc-119(+)] IV; unc-119(ed3) III* |
| GUN85 | *mewSi15[Pdpy-30::RFP:Mm_NECAP1 unc-119(+)] IV; unc-119(ed3) III* |
| GUN78 | *mewSi8[Pdpy-30::RFP:Mm_NECAP2 unc-119(+)] IV; unc-119(ed3) III* |
| GUN103 | *mewSi17[Pdpy-30::RFP:Ss_NECAP unc-119(+)] IV; unc-119(ed3) III* |
|  |  |

GFP-tagged AP2-α

| EG8012 | *oxSi254[Pdpy-30::APA-2::GFP unc-119(+)] II; unc-119(ed3) III* |
| --- | --- |
| GUN119 | *mewSi1[Pdpy-30::APA-2::GFP unc-119(+)] II; unc-119(ed3) III* |

YxxΦ cargo

| EG8608 | *oxSi484[Pvha-6::GFP:CD4:YASV unc-119(+)] II; unc-119(ed3) III* |
| --- | --- |

Heatshock TEV protease

| EG8531 | *oxSi883[Phsp-16.41::TEV(protease) unc-119(+)] II; unc-119(ed3) III* |
| --- | --- |

AP2-μ2-tev-site (HA)

| EG8524 | *unc-119(ed3) III; oxSi876[Papm-2::HA:APM-2:tev-site unc-119(+)] X* |
| --- | --- |
| EG8528 | *unc-119(ed3) III; oxSi880[Papm-2::HA:APM-2(E306K):tev-site unc-119(+)] X* |
| EG8526 | *unc-119(ed3) III; oxSi878[Papm-2::HA:APM-2(T160A):tev-site unc-119(+)] X* |
|  |  |

AP2-μ2-tev-site (FLAG)

| EG8525 | *unc-119(ed3) III; oxSi877[Papm-2::3xFLAG:APM-2:tev-site unc-119(+)] X* |
| --- | --- |

Figure 1

Starvation Assay

| EG6353 | *fcho-1(ox477::unc-119(+)) II; unc-119(ed3) III* |
| --- | --- |
| GUN101 | *fcho-1(ox477::unc-119(+)) II ncap-1(mew39[1.4kb deletion]) II* |
| GUN86 | *ncap-1(mew39[1.4kb deletion]) II* |
| GUN59 | *fcho-1(ox477::unc-119(+)) II mewSi2[Pdpy-30::RFP:NCAP1 unc-119(+)] II ncap-1(mew39[1.4 kb deletion]) II* |
|  |  |
|  |  |

Figure 2

AP2 localization and FRAP analysis in coelomocytes

| EG8012 | *oxSi254[Pdpy-30::APA-2::GFP unc-119(+)] II; unc-119(ed3) III* |
| --- | --- |
| EG6650 | *fcho-1(ox477::unc-119(+)) II oxSi254[Pdpy-30::APA-2::GFP unc-119(+)] II* |
| GUN98 | *fcho-1(ox477::unc-119(+)) II oxSi254[Pdpy-30::APA-2::GFP unc-119(+)] II ncap-1(mew39[1.4 kb deletion]) II* |
| GUN97 | *oxSi254[Pdpy-30::APA-2::GFP unc-119(+)] II ncap-1(mew39[1.4 kb deletion]) II* |
|  |  |

Cargo Assay

| EG8578 | *oxSi484[Pvha-6::GFP:CD4:YASV unc-119(+)] II; apm-2(ox546[W64X]) X oxSi876[Papm-2::HA:APM-2:tev-site unc-119(+)] X* |
| --- | --- |
| EG8579 | *fcho-1(ox477::unc-119(+)) II oxSi484[Pvha-6::GFP:CD4:YASV unc-119(+)] II; apm-2(ox546[W64X]) X oxSi876[Papm-2::HA:APM-2:tev-site unc-119(+)] X* |
| GUN65 | *fcho-1(ox477::unc-119(+)) II oxSi484[Pvha-6::GFP:CD4:YASV unc-119(+)] II ncap-1(mew39[1.4 kb deletion]) II; apm-2(ox546[W64X]) X oxSi876[Papm-2::HA:APM-2:tev-site unc-119(+)] X* |
| GUN66 | *oxSi484[Pvha-6::GFP:CD4:YASV unc-119(+)] II ncap-1(mew39[1.4 kb deletion]) II; apm-2(ox546[W64X]) X oxSi876[Papm-2::HA:APM-2:tev-site unc-119(+)] X* |

TEV/Phosphorylation assay

| EG8557 | *oxSi883[Phsp-16.41::TEV(protease) unc-119(+)] II; apm-2(ox546[W64X]) X oxSi876[Papm-2::HA:APM-2:tev-site unc-119(+)]* |
| --- | --- |
| EG8558 | *fcho-1(ox477::unc-119(+)) II oxSi883[Phsp-16.41::TEV(protease) unc-119(+)] II; apm-2(ox546[W64X]) X oxSi876[Papm-2::HA:APM-2:tev-site unc-119(+)] X* |
| GUN100 | *fcho-1(ox477::unc-119(+)) II; oxSi883[Phsp-16.41::TEVprotease unc-119(+)] II ncap-1(mew39[1.4 kb deletion]) II; apm-2(ox546[W64X]) X oxSi876[Papm-2::HA:APM-2:tev-site unc-119(+)] X* |
| GUN99 | *oxSi883[Phsp-16.41::TEVprotease unc-119(+)] II ncap-1(mew39[1.4 kb deletion]) II; apm-2(ox546[W64X]) X oxSi876[Papm-2::HA:APM-2:tev-site unc-119(+)] X* |
|  |  |

Figure 3

*C. elegans* NECAP transgenes

| EG8555 | *oxSi883[Phsp-16.41::TEVprotease unc-119(+)] II; apm-2(ox546[W64X]) X oxSi877[Papm-2::3XFLAG:APM-2:tev-site unc-119(+)] X* |
| --- | --- |
| EG8556 | *fcho-1(ox477::unc-119(+)) II oxSi883[Phsp-16.41::TEVprotease unc-119(+)] II; apm-2(ox546[W64X]) X oxSi877[Papm-2::3xFLAG:APM-2:tev-site unc-119(+)] X* |
| GUN96 | *fcho-1(ox477::unc-119(+)) II oxSi883[Phsp-16.41::TEVprotease unc-119(+)] II ncap-1(mew39[1.4 kb deletion]) II; apm-2(ox546[W64X]) X oxSi877[Papm-2::3xFLAG:APM-2:tev-site unc-119(+)] X* |
| GUN106 | *fcho-1(ox477::unc-119(+)) II oxSi883[Phsp-16.41::TEVprotease unc-119(+)] II ncap-1(mew39[1.4 kb deletion]) II; mewSi3[Pdpy-30::RFP:NCAP-1 unc-119(+)] IV; apm-2(ox546[W64X]) X oxSi877[Papm-2::3xFLAG:APM-2:tev-site unc-119(+)] X* |
| GUN91 | *fcho-1(ox477::unc-119(+)) II oxSi883[Phsp-16.41::TEVprotease unc-119(+)] II ncap-1(mew39[1.4 kb deletion]) II; mewSi15[Pdpy-30::RFP:Mm_NECAP1 unc-119(+)] IV; apm-2(ox546[W64X]) X oxSi877[Papm-2::3xFLAG:APM-2:tev-site unc-119(+)] X* |
| GUN93 | *fcho-1(ox477::unc-119(+)) II oxSi883[Phsp-16.41::TEVprotease unc-119(+)] II ncap-1(mew39[1.4 kb deletion]) II; mewSi8[Pdpy-30::RFP:Mm_NECAP2 unc-119(+)] IV; apm-2(ox546[W64X]) X oxSi877[Papm-2::3xFLAG:APM-2:tev-site unc-119(+)] X* |
| GUN95 | *fcho-1(ox477::unc-119(+)) II oxSi883[Phsp-16.41::TEVprotease unc-119(+)] II ncap-1(mew39[1.4 kb deletion]) II; mewSi17[Pdpy-30::RFP:Ss_NECAP unc-119(+)] IV; apm-2(ox546[W64X]) X oxSi877[Papm-2::3xFLAG:APM-2:tev-site unc-119(+)] X* |

Figure 5

Nerve ring microscopy

| GUN60 | *mewSi1[Pdpy-30::APA2:GFP unc-119(+)] I; mewSi2[Pdpy-30::RFP:NCAP1 unc-119(+)] II ncap-1(mew39[1.4 kb deletion]) II* |
| --- | --- |
| GUN61 | *mewSi1[Pdpy-30::APA2:GFP unc-119(+)] I; fcho-1(ox477::unc-119(+)) II mewSi2[Pdpy-30::RFP:NCAP1 unc-119(+)] II ncap-1(mew39[1.4 kb deletion]) II* |
| GUN62 | *mewSi1[Pdpy-30::APA2:GFP unc-119(+)] I; fcho-1(ox477::unc-119(+)) II mewSi2[Pdpy-30::RFP:NCAP1 unc-119(+)] II ncap-1(mew39[1.4 kb deletion]) II; apm-2(ox562[E306K]) X* |
| GUN53 | *mewSi1[Pdpy-30::APA2:GFP unc-119(+)] I; fcho-1(ox477::unc-119(+)) II mewSi2[Pdpy-30::RFP:NCAP1 unc-119(+)] II ncap-1(mew39[1.4 kb deletion]) II; apm-2(mew44[T160A]) X* |
| GUN55 | *mewSi1[Pdpy-30::APA2:GFP unc-119(+)] I; fcho-1(ox477::unc-119(+)) II mewSi2[Pdpy-30::RFP:NCAP1 unc-119(+)] II ncap-1(mew39[1.4 kb deletion]) II; apm-2(ox562[E306K]+mew46[T160A]*) X* |
| GUN56 | *mewSi1[Pdpy-30::APA2:GFP unc-119(+)] I; fcho-1(ox477::unc-119(+)) II mewSi2[Pdpy-30::RFP:NCAP1 unc-119(+)] II ncap-1(mew39[1.4 kb deletion]) II; apm-2(ox562[E306K]+mew47[R440S]*) X* |

* allele generated by CRISPR

Figure 6

TEV assay

| GUN128 | *fcho-1(ox477::cb-unc-119(+)) II oxSi883[Phsp-16.41::TEVprotease Cb_unc-119(+)] II ncap-1(mew39[1.4 kb deletion]) II; mewSi25[RFP:NCAP-1(A29D)*] IV; apm-2(ox546[W64X]) X oxSi877[Papm-2::3xFLAG:APM-2:tev-site Cb_unc-119(+)] X* |
| --- | --- |
| GUN135 | *fcho-1(ox477::cb-unc-119(+)) II oxSi883[Phsp-16.41::TEVprotease Cb_unc-119(+)] II ncap-1(mew39[1.4 kb deletion]) II; mewSi35[RFP:NCAP-1(S84N)*] IV; apm-2(ox546[W64X]) X oxSi877[Papm-2::3xFLAG:APM-2:tev-site Cb_unc-119(+)] X* |

* allele generated by CRISPR

Figure 6−figure supplement 1

Nerve ring microscopy

| GUN127 | *mewSi1[APA2:GFP] I; fcho-1(ox477::unc-119(+)) II mewSi24[RFP:NCAP1(A29D)*] II ncap-1(mew39[1.4 kb deletion]) II; apm-2(ox562[E306K])X* |
| --- | --- |
| GUN122 | *mewSi1[APA2:GFP] I; fcho-1(ox477::unc-119(+)) II mewSi31[RFP:NCAP1(S84N)*] II ncap-1(mew39[1.4 kb deletion]) II; apm-2(ox562[E306K])X* |

*allele generated by CRISPR

**B. Plasmids**

*C. elegans* NECAP transgenes

| pEP29 | Pdpy-30::TagRFP-T:worm_NCAP-1::unc-54UTR unc-119(+) cxTi10816 MosSCI |
| --- | --- |
| pEP41 | Pdpy-30::TagRFP-T:mouse_NECAP2::unc-54UTR unc-119(+) cxTi10816 MosSCI |
| pEP58 | Pdpy-30::TagRFP-T:mouse_NECAP1::unc-54UTR unc-119(+) cxTi10816 MosSCI |
| pEP71 | Pdpy-30::TagRFP-T:fungal(*Ss*)_NECAP::unc-54UTR unc-119(+) cxTi10816 MosSCI |
| pGH485 | Pdpy-30::TagRFP-T:NCAP-1(minigene)::unc-54UTR unc-119(+)  ttTi5605 MosSCI |
| pGH505 | [1-2]TagRFP-T:NCAP-1(minigene) |

Tissue culture pulldowns

| pGH500 | HaloTag_worm NCAP-1 pcDNA5frt |
| --- | --- |
| pGH501 | HaloTag_mouse NECAP1 pcDNA5frt |
| pGH502 | HaloTag_mouse NECAP2 pcDNA5frt |

Recombinant protein purification

| pGB19 | mouse β2(trunk) (1-591)_6xHis / mouse μ2 (E302K+thrombin site) |
| --- | --- |
| pGB21 | mouse β2(trunk) (1-591)_6xHis / mouse μ2 (+thrombin site) |
| pGB27 | HaloTag_TEV site_ mouse NECAP1_intein_CBD_6XHis |
| pGB28 | HaloTag_TEV site_ mouse NECAP2_intein_CBD_6XHis |
| pGB29 | HaloTag_TEV site_worm NECAP_intein_CBD_6XHis |
| pGB31 | mouse β2(trunk) (1-591)_6xHis / mouse μ2 (T156A+thrombin site) |
| pGB81 | HaloTag_TEV site_intein_CBD_6XHis |
| pEP82 | mouse AAK1 kinase domain (1-325) |
| pGB91 | HaloTag_TEV site_ mouse NECAP2(A32D)_intein_CBD_6XHis |
| pGB94 | HaloTag_TEV site_ mouse NECAP2(S87N)_intein_CBD_6XHis |
| pGH494 | 6XHis_HaloTag_linker_TEV site |
| pGH503 | 6XHis*_*HaloTag_linker_TEV site_ mouse NECAP2 |
| pGH504 | mouse AP2 α2(trunk) (aa 1-621)_HRV_GST/ rat AP2 σ1 (aa 1-142) |
|  |  |

**C. Oligonucleotides**

Identification of *ncap-1* mutants

| oGH678 | CGATAGAGAAGGCTTCAACACAC |
| --- | --- |
| oGH679 | AGGTATTCAGACATTTTTCAAATGAAAATCTAC |
| oGH680 | CAGTCAAAAAATGCGATAAAAGTACGG |
| oGH681 | GGACAGGAAATTTCAATAAATTAGCGATG |

*C. elegans* NECAP transgenes

| oEP366 | AACGGGCGGTAGTGGAGGCACTGGTATGGGAGATTACGAGAACGTTTTAATG |
| --- | --- |
| oEP367 | TATCACCACTTTGTACAAGAAAGCTGGGTCTAGAAATCTAATAAATTGCCAGACGTCG |
| oEP407 | GAGGAACGGGCGGTAGTGGAGGCACTGGTATGGAGGAGAGTGAGTACGAGTCTGTTCTGT |
| oEP408 | TCACCACTTTGTACAAGAAAGCTGGGTCTAGAACTGGACCCAGCCGGTG |
| oEP409 | GGAGGAACGGGCGGTAGTGGAGGCACTGGTATGGCGGCAGAGCTGGAATATG |
| oEP410 | TCACCACTTTGTACAAGAAAGCTGGGTCTAAAACTGGACCCAGTTAGATGGCTGTG |
| oEP391 | ACGTCGTGACTGGGAAAACCC |
| oEP392 | GCCAGGGTTTTCCCAGTCACGACGTTGATCATTGGCATGCTGAAATATTC |
| oGH526 | ATGGTTGTGTCGAAAGGCGA |
| oGH528 | ACCAGTGCCTCCACTACCGCCCGTTCCTCCTGTGCCACCTTTGTACAGTTCATCCATTCC |
| oGH698 | GGGGACAAGTTTGTACAAAAAAGCAGGCTCAAAAATGGTTGTGTCGAAAGGCGA |
| oGH731 | GTGACATTAAAGTCAAAAGCATCTCCTC |
| oGH733 | GGGGACAAGTTTGTACAAAAAAGCAGGCTCAAAAATGGGAGATTACGAGAACGTTTTAAT |
| oGH734 | GGGGACCACTTTGTACAAGAAAGCTGGGTTTAGAAATCTAATAAATTGCCAGACGTC |
| oGH736 | GAGGAGATGCTTTTGACTTTAATGTCAC |
| oGH738 | GCGGTAGTGGAGGCACTGGTATGGGAGATTACGAGAACGTTTTAATG |
| oGH1011 | TAGACCCAGCTTTCTTGTACAAAGTGGTGATA |
| oGH1012 | ACCAGTGCCTCCACTACCGCCCGTT |

Tissue culture pulldowns

| oGH953 | CATGCTTCCGCCGGTACCT |
| --- | --- |
| oGH954 | GTTTAAACCCGCTGATCAGCCT |
| oGH955 | GTGGAGGTACCGGCGGAAGCATGGGAGATTACGAGAACGTTTTAATG |
| oGH956 | GCTGATCAGCGGGTTTAAACTTAGAAATCTAATAAATTGCCAGACGTCG |
| oGH957 | GTGGAGGTACCGGCGGAAGCATGGCGGCAGAGCTGGAA |
| oGH958 | GATCAGCGGGTTTAAACTTAAAACTGGACCCAGTTAGATGGC |
| oGH959 | GTGGAGGTACCGGCGGAAGCATGGAGGAGAGTGAGTACGAGT |
| oGH960 | GCTGATCAGCGGGTTTAAACTTAGAACTGGACCCAGCCGG |

Recombinant protein purification

| oEP13 | TAATTAACCTAGGCTGCTGCCACC |
| --- | --- |
| oEP17 | AAGAAGGAGATATACATATGAAGAAGTTTTTCGACTCCAG |
| oEP18 | GGCAGCAGCCTAGGTTAATTACTGTACATTTGGAACGGGGC |
| oGB24 | CGCCGCCAGCCAATCTGCCCAGCCACCTGGCTGGTGATCTGGGACTGTTC |
| oGB26 | ATGAATAAGCLCTCCGATCATCATATGTATATCTCCTTCTTATA |
| oGB27 | GCATTTATGAAACCCGCTGCTAATTAACCTAGGCTGCTGCCACCG |
| oGB28 | ATGATCGGAGGCTTATTCATCT |
| oGB29 | GCAGCGGGTTTCATAAATGCCA |
| oGB33 | GGGCAGATTGGCTGGCGGCGAGAAGGCATCAAGTA |
| oGB34 | AGCAAGAGTCTGGTGCCGCGCGGCAGCGGTAAGCAGTCGATCGCCATTGATG |
| oGB35 | CTGCTTACCGCTGCCGCGCGGCACCAGACTCTTGCTTGTTTCATCAGCTGTG |
| oGB47 | TGAGATCCGGCTGCTAACAAAGCC |
| oGB48 | TTTAAGAAGGAGATATACATATGGCAGAAATCGGTACTGG |
| oGB49 | AGTGCATCTCCCGTGATGCAGAAATCTAATAAATTGCCA |
| oGB50 | AGTGCATCTCCCGTGATGCAAAACTGGACCCAGTTAGATGGC |
| oGB51 | AGTGCATCTCCCGTGATGCAGAACTGGACCCAGCCGGTGC |
| oGB52 | TGCATCACGGGAGATGCACT |
| oGB53 | GTTAGCAGCCGGATCTCAGTGGTGATGATGGTGATGTTGAAGCTGCCACAAGGCAGG |
| oGB174 | AGTGCATCTCCCGTGATGCAGCTTCCGCCGGTACCTCCAC |
| oGH338 | CATATGTATATCTCCTTCTTATACTTAACTAATATACTAAGATG |
| oEP642 | CCGATATCCACGGTTGGTGGCCCG |
| oEP643 | ACCAACCGTGGATATCGGGACTCAGAATGGCAACTGGACCAGC |
| oEP644 | TCTAGATACTTCGTCATCCGAATTGAAGATGGA |
| oEP645 | AATTCGGATGACGAAGTATCTAGAGTTGTCTGTCACACTCTCCACTGCG |
| oGH847 | CCAAACTGAAGGTCAAGGTGGTC |
| oGH848 | CCTTGACCTTCAGTTTGGTGCGC |
| oGH853 | TAATTAACCTAGGCTGCTGCCACCG |
| oGH1204 | GTTAATTAAAACAGATGCACGACGGTT |
| oGH1205 | GTGCATCTGTTTTAATTAACATGGAGGAGAGTGAGTACGAGT |
| oGH1206 | GCAGCAGCCTAGGTTAATTAGAACTGGACCCAGCCGG |
| oGH1227 | GGAAGTTCTGTTCCAGGGGCCCGGGTCCGGCATGTCCCCT |
| oGH1228 | GCCCCTGGAACAGAACTTCCAGGCCGGATCCGCCCTTCTT |
| oGH1231 | CATATGTATATCTCCTTCTTAAAGTTAAAC |
| oGH1246 | CCGCTGAGCAATAACTAGCATAAC |
| oGH1247 | CTAATGCAGGAGTCGCATAAGG |
| oGH1249 | GTTATGCTAGTTATTGCTCAGCGG |
| oGH1250 | TTATGCGACTCCTGCATTAGGCGCGAGGCAGGATCTCG |
|  |  |
|  |  |

Gene-specific target of crRNAs

| rEP360 | TGAAGTGTCTCGTAACAAGA |
| --- | --- |
| rGB156 | CAAATCACGTCTCAAGTGAC |
| rGB155 | TTGGGTGAAGTTCTAGCATC |
| rEP254 | CGGGCTGTCGAGGTTCCAGT |
| rEP676 | CACAAAATATCGAGAACTAT |
| rEP700 | CCCTGGCAACGCAATTGAGG |
|  |  |
|  |  |

CRISPR repair oligonucleotides

| oGB154 | TCCCATTGGTTCGcGAAGTGTCTCGTAACAAGATGaAAGTTAAGGTATTTCACTTGTCAC |
| --- | --- |
| oGB159 | TTCGTTACATTGGAcGATCGGGACTGTATGAAACtAGcTGCTAGAACTTCACCCAACCCT |
| oGB130 | GGAGCAGTCACAAATCACGTCTCAAGTtGCCGGCCAAATTGGATGGCGTCGGGAGGGTAT |
| oEP674 | TTCGCTATAAAATCCCTATTTTTCAGAGatGCgGACTGGAACCTCGACAGCCCGGCTTGG |
| oEP680 | CCGCCGATCGGAACCAGCGGTCATAAAGatGCgGACTGGAACCTCGACAGCCCGGCTTGG |
| oEP701 | GCCCGATCGATGCGCACCCTGGCAACGCAATTGAGGCcGTTTCgGATaacTCTaGATATTTTGTGATTCGTTTGCAG |
|  |  |
